# The Youth-Physical Activity Towards Health (Y-PATH) intervention: Results of a 24 month cluster randomised controlled trial

**DOI:** 10.1371/journal.pone.0221684

**Published:** 2019-09-13

**Authors:** Sarahjane Belton, Andrew McCarren, Bronagh McGrane, Danielle Powell, Johann Issartel

**Affiliations:** 1 School of Health and Human Performance, Dublin City University, Dublin, Ireland; 2 School of Computing, Dublin City University, Dublin, Ireland; 3 School of Arts Education & Movement, Dublin City University, Dublin, Ireland; 4 Carnegie School Of Sport, Leeds beckett University, Leeds, United Kingdom; University of New Mexico, UNITED STATES

## Abstract

Low levels of physical activity in youth are an issue internationally, with the age related decline in levels over the adolescent period of particular concern. This study evaluated a multi-component school-based intervention (Y-PATH: Youth-Physical Activity Towards Health), focused on halting the age-related decline in physical activity of youth in early adolescence. A cluster randomized controlled trial in 20 post primary schools (10 control, 10 intervention) was conducted. Data were collected from all 20 schools at baseline (2013), and 12 months (2014), and from 10 of these schools (5 intervention) at 24 months (2015). The setting was mixed gender post primary schools residing in the greater area of Dublin, Ireland. Principals from each school were asked to nominate one first year class group attending their school in September 2013 to participate in the study (N = 564). Intervention schools implemented the Y-PATH whole school intervention, comprising teacher component, parent component, and PE component; while control schools continued with usual care. The main outcome measure was accelerometer derived average minutes of daily moderate to vigorous physical activity (MVPA). Data were analysed from October 2015 –November 2017. At baseline 490 participants were assessed (mean age 12.78y ± .42). Results of the multilevel regression analysis confirmed that there was a significant time intervention effect, and this was predominantly contributed by the difference between control and intervention groups within females. Findings support the case for national dissemination of the Y-PATH intervention so that the knowledge learned can be translated to routine practice in schools.

## Introduction

Despite the known importance and associated benefits of regular physical activity (PA) for health, levels of PA decline dramatically during adolescence [1]. The most widely endorsed PA guideline stipulates that youth should accumulate at least 60 minutes of moderate-to-vigorous PA (≥ 60 min. MVPA) daily [2]. The Health Behaviour in School Aged Children study showed that in Ireland 31% of females, and 43% of males reported accumulating ≥ 60 min of MVPA daily at age 11 [3]. These low figures, which are relatively consistent with the findings of Woods et al.,[4] in a nationally representative Irish sample, declined to 20% of females and 36% of males by age 13 [3]. This decline reflects the critical period of transition of students from primary to post primary education, and is a key period for intervention.

The Centers for Disease Control and Prevention, in a review of evidence for PA promotion strategies [5] concluded that strategies with the potential for greatest reach, effectiveness and sustainability, such as enhanced PE in schools, should be given the highest priority for implementation. Consistent with recent findings [5,6], school-based physical education (PE) was highly recommended as an intervention strategy [7]. A recent review [8] highlighted that comprehensive school based interventions with components targeting both physical activity and health education appear most effective. Van Sluijs *et al*. [9] in a similar review found that there was strong evidence showing that school-based interventions with a family or community component can increase PA in adolescents (defined as ≥ 10 years). This is supported by the findings of Kriemler et al. [10], who identify school-based application of multi-component intervention strategies as the most promising and consistent strategy for PA intervention with youth, and those of Krahnstoever Davison et al. [11] who demonstrated the importance of peer and family support for PA participation in youth.

The World Health Organisation [12] emphasised the need for research to generate sound knowledge on models of successful intervention in PA. The Waters et al. [13] review indicates that testing short-term, behaviourally focused school-based interventions for 6–12 year old children is no longer warranted, but that the gap in research now relates to effective interventions for adolescents. Van Sluijs et al. [9], in their systematic review of youth PA interventions identified short follow up (less than six months) as a limitation across the studies reviewed. The Salmon et al. [14] review also highlighted short-term follow-up as an issue, and recommend that future studies must include a follow-up of at least 1–2 years. Similarly Dobbins et al. [15] in a Cochrane systematic review of school-based PA programmes, highlighted the need for follow up of PA interventions so that long-term impact can be determined. This lack of long-term follow up along with poorly powered designs as highlighted by Waters at al. [13], may well explain findings of one recent systematic review [16], where a low overall effect for interventions on increasing minutes of MVPA amongst children and adolescents was cited. Waters et al. [13] recommend larger, sustainable and longer-term studies, guided by theories such as the socio-ecological model, powered to detect the small changes that are likely to be found.

Following the recommendations and targeted strategies alluded to above, the Y-PATH (Youth-Physical Activity Towards Health) programme was developed as a research-informed and evidence-based multi component school-based intervention [17]. Y-PATH aims to improve PA levels of adolescents through (i) education about the importance of PA for health and the dangers of sedentary behaviour, (ii) increasing proficiency in basic movement skills essential for participation in PA known as fundamental movement skills (FMS), and (iii) improving levels of self-efficacy motivation, regulation, and empowerment. In addition to targeting the adolescent at the individual level, Y-PATH also extends to the whole school by targeting the teachers and parents within the intervention. The Y-PATH programme is the first evidence-based intervention of its kind in Ireland, and was developed following cross-sectional exploratory research [18], using a social-ecological framework, and based on the Youth Physical Activity Promotion Model [19], with subsequent development of the teacher education element of the intervention based on Self-Determination Theory [20,21]. Central to the development of the Y-PATH intervention at all stages was the need for the intervention to be cost-effective, time-efficient for teachers, and scalable to a national level.

Previous research on the Y-PATH intervention has included a one-year two school exploratory trial [21] and a one-year 20-school cluster randomized control trial [22]. In both of these trials, an intervention effect was found for FMS proficiency (measured using the Test of Gross Motor Development—Second Edition) [23] (Ulrich, 2000), while an intervention effect for PA (measured by self-report and objective accelerometry) was found in the exploratory trial only [21]. The purpose of the present study is to build on this previous work, and address the research gaps alluded to above, to investigate the effect of participation in the Y-PATH intervention over a two-year period on objectively measured MVPA levels of young people.

## Methods

### Study design, setting and participants

The Y-PATH cluster randomized controlled trial targeted first year post primary students (12–13 years old) attending post primary education within a particular Irish geographical region. Inclusion criteria for post primary schools in this study were that a) schools have a qualified PE teacher on staff, b) first year students attending the school were timetabled for a minimum of 70 minutes of PE weekly, c) schools were mixed gender and situated in the greater area of a large Irish city. All mixed-gender schools in the particular Irish geographical region (n = 104) were invited to express interest in participation in the study if they met the above inclusion criteria. This trial was registered retrospectively with the ISRCTN (International Standard Registered Clinical/soCial sTudy Number), and the trial record is available at http://www.isrctn.com/ISRCTN20495704.

Sample size estimation was carried out by considering the 12% of Irish adolescents estimated to meet the 60-minute daily MVPA guideline [4] nationally in Ireland. A total of 18 schools (9 per arm, with an average of 27 participants per school) were estimated to provide at least 80% power at a 5% level of significance (2-sided) to detect a 20% difference (with an intraclass correlation of 0.1) in the proportion of children meeting the 60-minute MVPA guidelines at 12 months. To allow for attrition, a further 2 schools per arm (with 27 students per school) were required, increasing the targeted sample to 20 schools. Principals of 26 schools returned expressions of interest, screening of these schools highlighted that 22 schools met the inclusion criteria, all 22 schools were recruited to participate in the study. One first year class group from each school was randomly selected by the school principal to participate. Two schools subsequently withdrew from the study prior to commencement due to changes in staffing (PE teacher and principal), reducing numbers to 20 overall.

Randomization was carried out at the school level rather than at the student level, to minimize the possibility of students or teachers in the control groups being influenced by intervention group participants. The 20 recruited schools were pair-matched prior to baseline testing based on the following criteria: socioeconomic status (disadvantaged, non-disadvantaged, and fee paying), school size (small 0–299 students, medium 300–599 students, large >599 students), and facilities (school hall, size of hall, basketball courts etc.). One school from each pair was then randomly allocated by the study principal investigator to the control group (and the other to the intervention group) using a manual number generator in blocks of 1:1, prior to the commencement of baseline testing.

**Fig 1:**
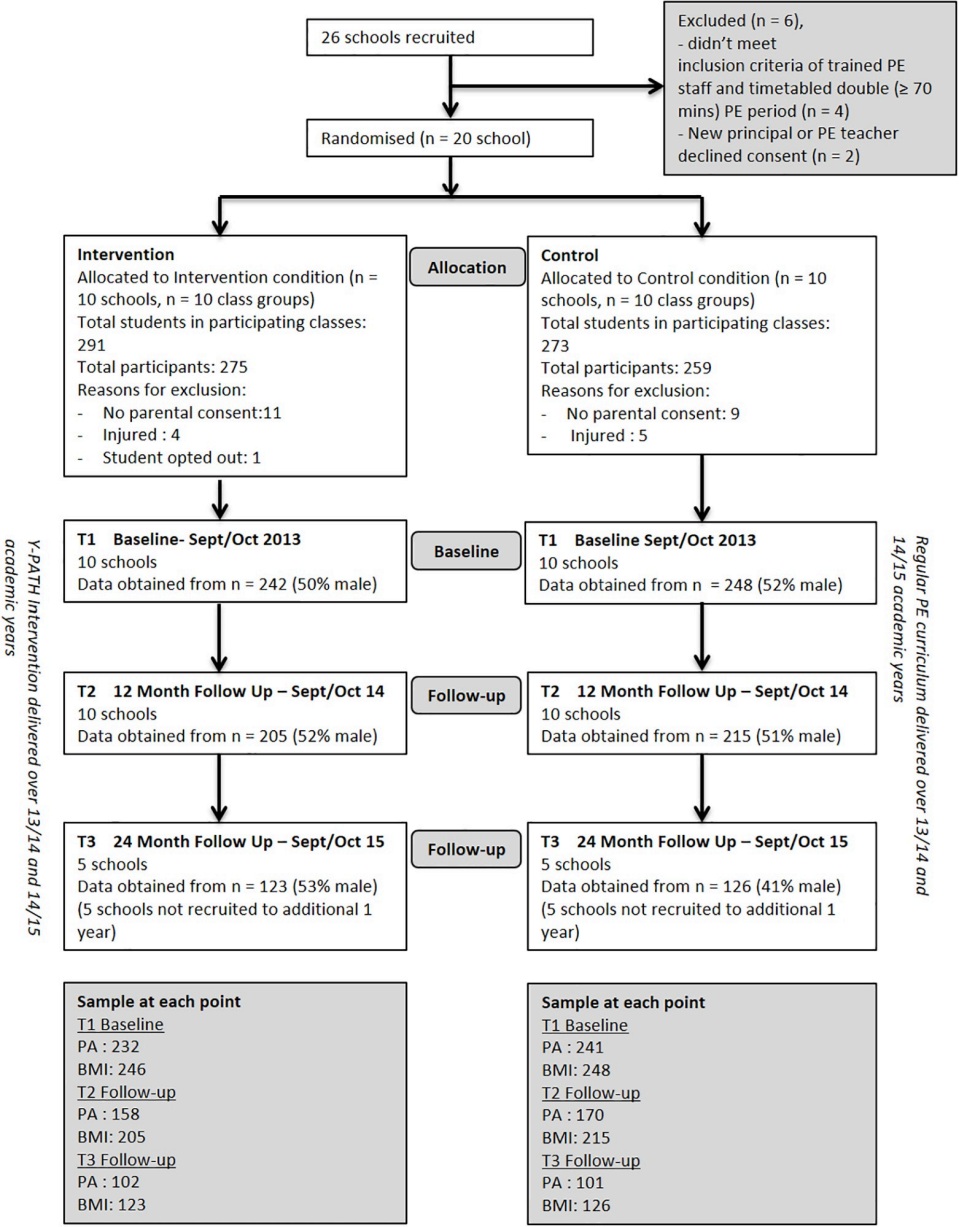
Flow of participants through the study.

Outcome assessments were conducted with students in all 20 schools at baseline (T1, September-October 2013), at 12 months follow up (T2, September-October 2014). All schools were invited to remain involved in the study for a further 12 months. Ten of these schools (5 intervention and 5 controls) consented to remain involved in the study, with the remaining ten unable to continue beyond the original 12-month commitment for a variety of reasons (including change in principal or PE teacher, and commitments already made to other PA initiatives for the following 12 months). As such, outcome assessment was conducted with this sub-sample of 10-schools at 24 months follow up (T3, September-October 2015). Principal consent, opt-in written parental consent, and participant assent were required prior to data collection. All participants were free to withdraw from the research at any stage. Full approval for this study was granted by the Dublin City University Research Ethics Committee (DCUREC/2010/081). CONSORT guidelines were followed to ensure no bias was observed in this study. Fig 1 documents the flow of participants through the study. The lower numbers at T2 compared to T1 are explained by i) children’s absence from school on the day of testing, ii) children choosing to withdraw from the study, and iii) injury/illness that prevented them from completing the protocol. The fewer schools involved at T3 explains in large part the lower number at this time point, along with reasons i) to iii) outlined above.

### Intervention

#### Guiding principles

**Fig 2:**
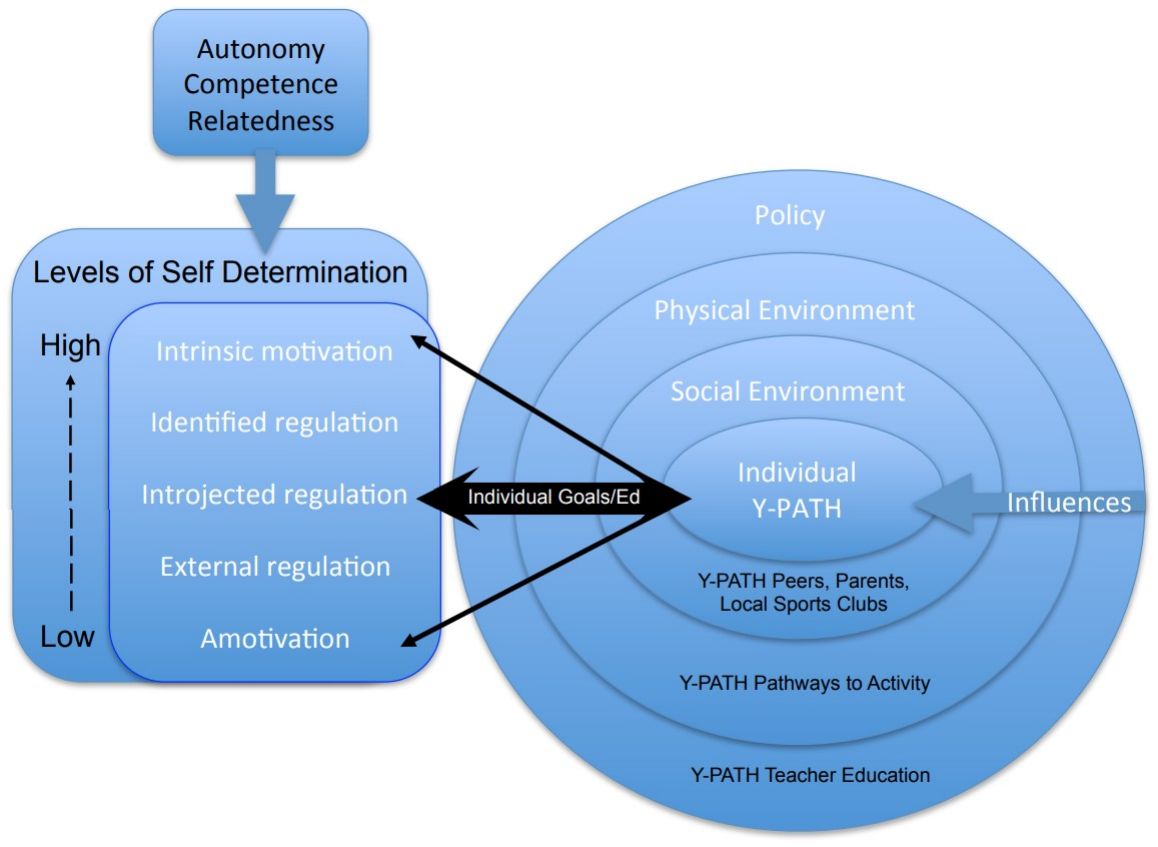
Theoretical framework of the Y-PATH intervention.

The theoretical framework driving the Y-PATH intervention is shown in Fig 2. Full detail on the development of the Y-PATH intervention, and of the various components involved in the intervention, are given in Belton et al. [18]. The guiding principles of the intervention are given below:

1. The first experience of PE for the students at second level school will be Health-Related Activity, with a focus on PA participation (move from PE being associated with a specific activity or sport, to being associated with learning to be active)
2. PE lessons will focus on maintaining MVPA levels, and improving students’ attitude towards PA, self-efficacy and FMS levels.
3. The climate in PE lessons will be motivational; all students learn that they can be active, experience a range of choice, and learn to challenge themselves and experience success within their own parameters (focus on attitude and self-efficacy).
4. Parents/guardians and non-specialist PE teachers targeted as role models that can have a significant influence on students’ attitudes towards PA participation (move from traditional notion of PE teacher being the person in the school with sole responsibility for health and PA).

#### Components of the Y-PATH intervention

The Y-PATH intervention is a whole-school multi-component intervention programme, aimed at reducing the age-related decline of MVPA in adolescents. The different components target students, teachers and parents, with a PE component, a whole-school teacher component and a parent component.

**PE Component:** Y-PATH PE has a strong focus on physical literacy development (developing student motivation, self-confidence, FMS mastery, physical fitness, and Health-Related Activity knowledge) within the PE class, with the school’s qualified PE teacher trained to deliver Y-PATH PE over the full academic year. Crucially, Y-PATH PE does not add to the existing teaching requirements of the PE teacher, rather offers a renewed structure and emphasis through which to deliver the existing state curricula for PE at Junior Cycle. Y-PATH PE fully upholds the existing Department of Education and Skills’ Junior Cycle Physical Education Curriculum [24], but changes the focus, direction and philosophy of delivery.

PE teachers in the intervention condition received four hours of Y-PATH Continuing Professional Development on the implementation of the Y-PATH-PE element, prior to the commencement of the academic school year. As part of this element of the intervention the PE teachers receive a set of six targeted lesson plans to be delivered by the PE teacher to their class groups for the first six weeks of the academic year in first year, and a second set to be delivered for the first six weeks of the second academic year. These lessons focus heavily on motivational climate, integrating health related activity core knowledge through fun and engaging practical lessons, with an emphasis on FMS proficiency throughout. For the remainder of the school year, the teachers are given a set of resource cards as teaching prompts to enable them to integrate a Health-Related Activity and FMS focus within the other core PE content areas (Gymnastics, Dance, Aquatics, Games, Outdoor and Adventure Activities, and Athletics) while teaching for a motivational climate. Y-PATH PE is supported by a student ‘PA journal’ that teachers are asked to integrate as part of regular PE class, so that students learn to track their PA behaviours and identify ways they could improve their PA levels. In addition, a local ‘PA directory’ that contains information and contact details for a range of youth sport and PA clubs in the local community is provided, to assist PE teachers in linking students with PA opportunities beyond the school.

**Whole-School Component:** The whole-school component included two ‘PA Promotion’ workshops for teachers delivered by a Y-PATH-trained facilitator, as well as the development and implementation of a school ‘charter’ for physical activity with specific targets agreed by the school community. All teachers within the school are encouraged to be ‘active role models’ for students.

**Parent Component:** This included an information evening delivered by a Y-PATH-trained facilitator, and a parents’ PA information leaflet distributed periodically through the school newsletter. Both the information evening and the information leaflets highlight key strategies for promoting PA beyond the school environment which are discussed with parents and emphasized periodically.

Schools allocated to the intervention condition were asked to implement the whole-school Y-PATH intervention over the full academic year, the 5 intervention schools that remained in the study for the second academic year were asked to implement the intervention for this second year also. Control schools were asked to continue with usual care, without any researcher input over the academic year. Usual care in this context consisted of regular delivery of the Irish Junior Cycle PE curriculum, and their broader school curricula.

### Data collection

Measures taken in this study were accelerometer measured MVPA, and body mass index (BMI). The measurements are detailed below, and were taken by trained research assistants who visited the schools at each of the three time points (T1, T2, and T3). Research assistants were not blinded to control or intervention condition assignment as it was not possible given the nature of the intervention (for example Y-PATH teaching posters are displayed on school walls as part of the intervention).

Accelerometer data was used to derive the primary outcome measure, mean student duration (minutes) of MVPA per day. Participants were asked to wear an Actigraph accelerometer (models GT1M, GT3X or GT3X+; Actigraph LLC, Pensacola, FL) on an adjustable elasticated belt above the iliac crest of the right hip. Participants were asked to wear the accelerometer during all waking hours, with the exception of water-based activities such as showering and swimming, and contact sports deemed unsafe for accelerometer wear (e.g. rugby) for a total of nine consecutive days. Accelerometers were set to record using a 10-second epoch [25]. A number of strategies were employed to maximize wear-time compliance [26]: students were met in the morning of each school day to ascertain compliance with the wear instructions; an optional twice daily SMS reminder text was sent to students before school, and in the afternoon immediately after school; a specific teacher in each school checked whether or not participants were wearing their monitors during each school day; students were advised to place reminders to wear monitors in noticeable areas in their homes; and students who were compliant with the wear-time inclusion criteria, entered a class draw for a €20 sports voucher (per class).

In line with previous studies [25,27], the minimum number of valid days required for inclusion in accelerometer data analysis was two days [28], a day was deemed valid (and therefore included in analysis) if there was a minimum of 8 hours recorded wear-time per day [29], and monitor non-wear was defined as ≥20 consecutive minutes of zero counts. Any counts below zero and above 15,000 were excluded, due to biological implausibility [30]. The mean daily minutes spent in moderate-to-vigorous physical activity (MVPA) were estimated using the validated cut points, derived by Evenson et al. (2008) for adolescents in this age group: MVPA ≥ 2296 counts/min.

Standing height was measured using a portable stadiometer (Leicester Height Measure) in centimetres (cm) to the nearest two decimal places. Weight was measured using a portable calibrated scales (SECA) in kilograms (kg) to the nearest 0.5kg. BMI was calculated using the formula weight(kg)/height^2^(m^2^). Participants completed the measurements in light clothing without shoes.

### Statistical analysis

Multilevel linear regression analysis was used to examine the effect of the Y-PATH intervention on average minutes of MVPA (Total MVPA). A three level multilevel structure was proposed with random intercepts, where time (Level one), pupils (Level two) and schools (Level three) served as the grouping variables, where time was treated as a fixed effect in the model but was also incorporated as a random slope effect (repeated measure) in the residual component. The structure is outlined in Eqs 1–5. All fixed effect interactions were examined. The repeated measures component was analyzed for unstructured, unstructured heterogeneous Eq (5), autoregressive, autoregressive heterogeneous and compound symmetry variance structures.

$$\begin{array}{cc} \;Y_{ijk}= & \beta_{0}+\beta_{1}t_{ij}+\beta_{2}g_{ij}+\beta_{3}TX_{ij}+\beta_{4}t_{ij}{}^{2}+\beta_{5}g_{ij}TX_{ij}+\\ & \beta_{6}g_{ij}t_{ij}+\beta_{8}t_{ij}TX_{ij}+\beta_{6}g_{ij}t_{ij}TX_{ij}+\beta_{7}BaseCovariate_{ij}+\\ & \beta_{8}BaseCovariate_{ij}TX_{ij}+\;U_{0j}+V_{oij}+\varepsilon_{ijk} \end{array}$$ (1)

With

$$U_{0j}\;=\;N(0,{\sigma_{u0}}^{2})$$ (2)

And

$$V_{1ij}\sim N(0,{\sigma_{v0j}}^{2})$$ (3)

And

$$\varepsilon_{ijk}\;\sim \;N(0,R)$$ (4)

$$R\;=\;(\begin{array}{ccc} R_{1} & 0 & 0\\ 0 & \ddots & 0\\ 0 & 0 & R_{n} \end{array})$$

And

$$R_{sub}\;=\;(\begin{array}{ccc} {\sigma_{\varepsilon_{11}}}^{2} & {{\rho_{12}\sigma }_{\varepsilon_{12}}}^{} & {{\rho_{13}\sigma }_{\varepsilon_{13}}}^{}\\ {{\rho_{12}\sigma }_{\varepsilon_{12}}}^{} & {\sigma_{\varepsilon_{22}}}^{2} & \rho_{23}{\sigma_{\varepsilon_{23}}}^{}\\ {{\rho_{13}\sigma }_{\varepsilon_{13}}}^{} & \rho_{23}{\sigma_{\varepsilon_{23}}}^{} & {\sigma_{\varepsilon_{33}}}^{2} \end{array})$$ (5)

Where *t*_*ij*_, *g*_*ij*_ and TX_ij_ are time, gender and treatment for student i from school j at time point k for n subjects, $\sigma_{\varepsilon_{kk^{\prime }}}\;=\;\text{covariance}\;\left(\varepsilon_{\text{k}},\;\varepsilon_{\text{k}’}\right)$ (k ≠ k’) and ρ_*kk'*_ = correlation (ε_k_, ε_k’_).

The model outlined in Eq 1 also had higher order time covariates incorporated to examine the possibility of a non-linear time effect on MVPA. Heterogeneity of treatment effects was examined by incorporating an additional interaction between treatment and the baseline BMI and baseline MVPA covariates. Regression coefficients for the group variables (where ‘0’ indicated Control schools, and ‘1’ indicated Intervention schools) reflected average differences in the outcome variable over time adjusted for baseline outcome values, timing of follow-up measures, and a priori covariates known to moderate MVPA (gender and BMI) over 3 time periods. To determine the time points at which any intervention effects occurred at (T1 (0), T2 (1), or T3 (2)), post-hoc stratified analyses comparing the estimated marginal means of the interaction variables were performed for the Intervention and the Control groups, and comparisons were made with t-tests using Satterwhaite degrees of freedom. Random Intercepts were assessed for significance using the Wald statistic with statistical significance set at p<0.05. The covariance structure of the mixed model outlined in Eq 1 was evaluated by assessing the Akaike (AIC) information criterion and Bayesian information criterion (BIC). Analyses were performed using SPSS software version 24 (IBM Corporation).

## Results

**Fig 3:**
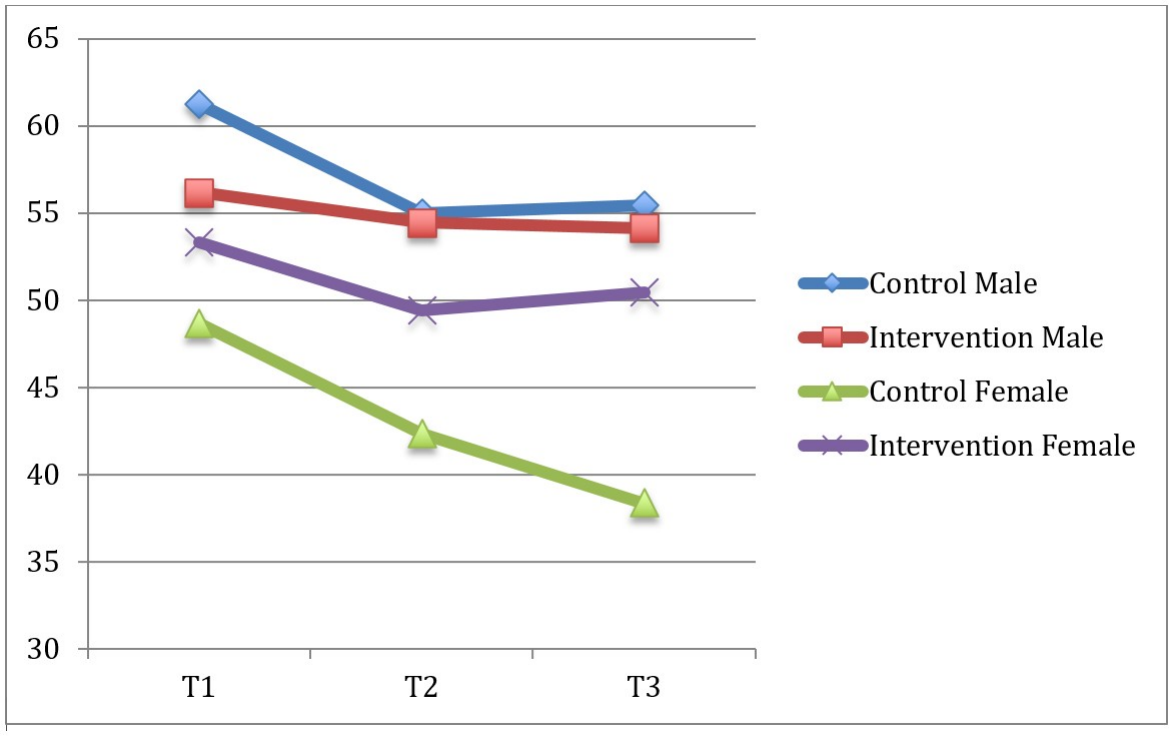
MVPA (minutes/day) across time and gender for control and intervention groups.

A total of 490 participants from 20 schools were involved in this research at baseline (T1), this dropped to 420 participants from 20 schools at 12-month follow-up (T2), and to 249 participants from 10 schools at 24-month follow-up (T3). Baseline characteristics of the participant sample by gender and intervention condition are given in Table 1. An independent samples t-test revealed no significant differences in age or BMI between participants that met or did not meet the accelerometer inclusion criteria. Minutes of MVPA by gender and intervention condition across the three time points are given in Table 2, and shown graphically in Fig 3.

**Table 1:** Baseline characteristics (mean ± standard deviation) by gender and intervention condition.

	Control		Intervention	
	Male	Female	Male	Female
Age (years)	12.81 ± .44	12.8 ± .41	12.8 ± .42	12.79 ± .4
BMI (kg/m^2)^	19.34 ± 3.15	20.29 ± 2.81	20.01 ± 3.06	20.83 ± 3.45
MVPA (mins/day)	61.24 ± 24.97	48.68 ± 22.41	56.18 ±2 0.54	53.34 ± 18.77

**Table 2:** Sample size and MVPA (minutes/day) at each time point by gender and intervention condition.

	Control				Intervention			
	Male		Female		Male		Female	
	N	Mins MVPA	n	mins MVPA	N	mins MVPA	n	mins MVPA
T1	123	61.25	118	48.68	112	56.18	118	53.34
T2	80	54.99	90	42.33	76	54.45	82	49.44
T3	40	55.46	61	38.35	48	54.12	54	50.47

An Autoregressive (AR) covariance structure was found to have the lowest AIC and BIC. The random intercept for School was found to be marginally insignificant (21.439, p = 0.051, Wald Z = 1.953), and the AR diagonal (233.255, p<0.001, Wald Z = 19.467) and the AR ρ (.125, p = 0.01, Wald Z = 2.574) of the error term were found to be significant. In this model the intercept term at a student level was excluded, as it was described fully by AR covariance structure. The higher order powers of time, the interaction between baseline BMI and treatment were excluded from the final model as they were highly insignificant and they had no direct impact on lower order model terms. Additionally, including baseline BMI would have reduced the data volume by over 10% due to the missing values.

The final parameter estimates for the fixed effects of the final model choice are shown in Table 3, and similarly the Type III test effects for the final interaction effects are shown in Table 4.

**Table 3:** Parameter estimates of main fixed effects.

Parameter	Est.	S.E.	D.F.	t	p	C.I.
**Intercept**	24.961	3.495	80.134	7.141	<0.001**	(18.005,31.918)
**Time**						
Time (1v2)	-0.932	2.278	772.062	-0.335	0.738	(-6.404,4.539)
Time (1v3)	-1.185	2.797	480.827	-0.424	0.672	(-6.681,4.311)
**Gender (M/F)**	5.982	3.09	771.32	1.936	0.053	(-0.084,12.047
**Intervention (C/T)** For Gender = Female and time = 4	-11.842	4.690	80.919	-2.525	0.014*	(-21.175,-2.509)
Gender *Intervention	4.369	4.422	772.955	0.988	0.323	(-4.321,13.049)
Time*Intervention						
Time 1 * Male	-3.352	3.935	739.160	-0.852	0.395	-(11.076,4.373)
Time 3 * Male	0.841	4.009	506.500	0.210	0.834	(-7.0358, 8.718)
Time* Intervention						
Time 1 * Control	7.487	3.753	771.995	1.995	0.046*	(0.121,14.853)
Time 3 * Control	1.645	3.763	498.101	0.437	0.662	(-5.747,9.038)
Gender*Intervention*Time						
Time 1 * Male*Control	-2.212	5.470	770.614	-0.404	0.686	(-12.951,8.526)
Time 3 * Male*Control	-3.879	5.613	538.746	-0.691	0.490	(-14.906,7.147)
**Baseline MVPA**	0.501	0.045	505.010	11.112	<0.001**	(0.409,0.586)
Intervention*Baseline MVPA	0.103	0.057	468.847	1.800	0.072	(-0.009,0.216)

* Implies significance at the α = 0.05,

** Implies significance at the α = 0.01

**Table 4:** Type III analysis of interaction effects.

Parameter	F	D.F.	p
Time * Intervention	4.537	(2, 582.684)	0.011*
Gender * Intervention	0.902	(1,403.048)	0.343
Gender*Time	1.634	(2,592.168)	0.196
Time * Intervention*Gender	0.239	(2,592.168)	0.787
Baseline MVP * Intervention	3.240	(1,486.847)	0.072

*Implies significance at the α = 0.05

Post hoc analysis on the group comparisons of the intervention interaction with time are outlined in Table 5.

**Table 5:** Post hoc contrast test analysis for significant interaction effects.

Comparison	Estimated Difference	d	p	C.I.
Baseline Control Baseline Intervention	-3.277	0.205	0.443	(-11.773,5.217)
Post Control vs Post Intervention	-9.657	0.604	0.03*	(-18.364,-0.952)
Post Female control vs Post Female Intervention	-11.842	0.742	0.014*	(-21.175,-2.509)
Post Male control vs Post Male Intervention	-7.476	0.465	0.143	(-17.519,2.572)

* Implies significance at the α = 0.05,

## Discussion

The purpose of this study was to investigate the effect of participation in the Y-PATH intervention over a two-year period on objectively measured MVPA levels of young people. Data collected and analysed in this study confirm the well-known and frequently observed gender disparity in physical activity levels in youth [31,4,32] and the frequently reported age related decline in physical activity [31,4,32]; with males average daily MVPA dropping by 5.79 minutes over the two year period, and females average daily MVPA dropping by 10.33 minutes over the same period (the decline further exaggerating the gender disparity). Study findings provide some support for the efficacy of the Y-PATH intervention. After 24 months, the intervention was effective in maintaining MVPA levels specifically in females in the intervention condition, while control participants levels significantly declined. The findings suggest that implementing the Y-PATH intervention in post primary schools may have the potential to help address the age-related decline in female MVPA levels.

This study evaluating the Y-PATH intervention builds upon and supports the positive findings demonstrated in previous research [27,22] for the efficacy of the Y-PATH programme. Fig 3 graphically presents the trends in average daily minutes of MVPA across gender and time for control and intervention conditions. From this graph the downward trend in MVPA over time is apparent for all groups, with the biggest decline evident for males in the control condition (where a higher level of MVPA at baseline was observed), and females in the control condition. Results of the mixed model analysis confirmed no main effect for BMI, but showed a significant time*intervention effect which was predominantly contributed by the impact of the intervention on females. The significant interaction effects found within the data are presented in Table 4. The post hoc analysis in Table 5 demonstrates how the interaction between time and intervention occurs. One can see that there was no significant difference between control and intervention at baseline, but a significant difference is evident between the control and intervention at 24-months; with control participants demonstrating a significant decline in minutes of MVPA over the course of the intervention period. Findings are consistent with the results of the Physical Activity 4 Everyone cluster randomised trial in Australia [33], a trial similar to that reported in the current study. Sutherland et al. [33] evaluated the 24-month intervention effect of the multicomponent school based Physical Activity 4 Everyone intervention in disadvantaged schools (children aged 12 years at baseline). Sutherland et al. [33] reported that the intervention was effective in increasing adolescents minutes of MVPA, and concluded that implementing the intervention may have the potential to slow the age related decline in physical activity.

This finding is also consistent with the results of the HEalth in Adolescents (HEIA) study in Norway [34], which evaluated the intervention effect of the HEIA 20-month multicomponent school based intervention (children aged 11 years at baseline), and similarly found a stronger effect in females. The reason hypothesized by Grydeland et al. [34] for this gender effect, was that the intervention while not developed specifically for females, it was developed bearing low levels of female physical activity in mind. The same is true for Y-PATH PE. Moreover, the pedagogical focus within Y-PATH PE was to move PE class towards motivational climate, with an emphasis on student mastery rather than competition. Considering the consistent message from research through the years highlighting competition as a barrier to PE participation and enjoyment for females particularly [35–37], it may well be this pedagogical focus which explains the stronger finding for females in this study.

The positive finding in terms of females who experienced the Y-PATH intervention in the current study are particularly encouraging when we consider findings of a recent systematic review and meta-analysis of the effectiveness of school based physical activity interventions in adolescent girls [38]. These authors [38] concluded, concluded, based on the small intervention effects observed across studies reviewed, that changing PA behaviour of adolescent females through school based interventions is challenging. The authors did go on to state however that multi-component interventions, and interventions based on theory may provide the strongest chance of positively impacting PA behaviour of this at risk cohort [38]; the fact that Y-PATH meets both of these criteria may well explain the positive result found in this study.

Similarly in another systematic review [39] it was concluded that PA was a sustainable outcome from interventions in children and adolescents, with interventions such as Y-PATH which are longer than 1 year in duration and based on a sound theoretical model more likely to achieve sustained impact. This is consistent with the recent work of Meyer et al. [40] who advocated the need for longer sustained intervention programmes for sustained impact on health outcomes. In a recent systematic review of school based interventions aimed at increasing physical activity (and fitness) levels of youth [10], the authors conclude that school based application of multi-component interventions was the most consistent and promising strategy of those reviewed. The results presented in this paper on the outcomes of the Y-PATH intervention add further support to the conclusions’ of Kriemler et al., [10], and further point to the important role of school based multi-component interventions as a strategy for increasing physical activity levels of youth.

The importance of targeted strategic intervention development and evaluation is also highlighted in the positive findings of this study. Y-PATH was developed based on careful consideration of the context and situation of the specific target cohort of adolescent Irish youth [18] to ensure it could meet their needs, and would work within the broad school and PE environment, specific to the Irish context. The Medical Research Council’s [41] advice on the development and evaluation of complex interventions was respected, with initial pilot, feasibility and evaluation research [18,27,22], followed by the two-year definitive trial reported in this paper. The partnerships developed over the course of this work, as documented in Belton et al. [17], have included local and national stakeholders to ensure that the next step of the Medical Research Council guidelines could be followed; the implementation phase where intervention findings can be translated into routine practice. The need for an intervention such as Y-PATH to address physical activity problems of Irish youth has been very well explicated in recent years at a national level. In 2016 Ireland published the first time a National Physical Activity Plan [42], with interagency agreement between Government departments. Action area two defined in the National Physical Activity Plan focuses on children and young people, with the specific target being that *‘Children and young people learn the necessary skills for confident engagement with physical activity and will have opportunities to adopt an active way of life’* (pp 17; [42]. The government Departments which collaborated in the development of this plan highlighted that being active was central to health of children and young people, with key emotional, social and cognitive benefits. They further identified the importance of the school setting, a location where *‘the knowledge*, *skills and behaviours which are likely to enhance lifelong engagement in physical activity and good health’* should be developed (pp 17; [42]). Of key note in this document was the recognition of the role the school system should play, and in particular quality PE, in enabling children to lead physically active lives through the mastery of fundamental skills, and the acquisition of relevant knowledge and positive attitudes.

These elements of quality PE recognised in the National Physical Activity Plan [42], are consistent with those highlighted in the concept of ‘physical literacy’, a term which is growing in use in recent years. Physically literate people are those that move well, move often, and move confidently in a variety of situations to the benefit of their health [43–45]. Through the research carried out in the development of Y-PATH, the importance of positive attitudes, self-efficacy, motivation, and FMS ability to enable youth to be active was highlighted [18]; and the programme was built to satisfy these physical literacy needs. Y-PATH is a research-informed and evidence-based intervention programme [18,27,22], which meets many of the goals and recommendations of Irelands National Physical Activity Plan [42] as highlighted above. Perhaps more importantly when national implementation is considered as a target however, the Y-PATH intervention was also developed with and for schools, and specifically to uphold and support the core tenets of the national PE curriculum for youth this age [46,24,18]. The findings of this study support that the Y-PATH intervention which was developed to meet the specific needs of Irish youth, can play a role in helping to alleviate the known age related decline in PA that happens over this critical adolescent period. More recent research on the Y-PATH programme has seen the development of a targeted and sustainable national implementation plan, with multi agency support to ensure that the knowledge learned through this research can be translated into routine practice in Irish schools [17]. In September 2018, the Irish Heart Foundation committed to national dissemination of Y-PATH, with a target of reaching every school in the country by 2021. Dissemination is being led by the Irish Heart Foundation, in partnership with Dublin City University, and supported by University College Cork, Sport Ireland, and the Professional Development Service for Teachers (state body with responsibility for provision of professional development to teachers in Ireland)[17]. Further research to confirm the findings of this study on a larger scale, and track fidelity as the programme is scaled up, is warranted as the programme is rolled out to more schools nationally.

### Strengths and limitations

Following recommendations of other authors this evaluation involved interim measurements over the rollout period [15], and with retention follow-up of at least one year [9,47]. As recommended in these review papers [15,13,9], an objective measure of PA (accelerometry) was employed as the primary outcome measure, and as advocated by Kriemler et al., [10], measurement wasn’t restricted to the school day but included total PA. The choice of two days as our wear criterion may be considered a limiting factor, and in line with the discussions presented in Rich et al. [28] was chosen to strike a balance between reliability of estimates and sample size. The poor compliance with accelerometer wear criteria must be acknowledged as a limitation however, with 86% meeting the 2 day criterion at T1, 60% at T2, and 82% (of the reduced sample of 10 schools) at T3. This issue is common across intervention studies such as that presented in this paper [48]. The smaller sample size at T3 due to the limited number of schools participating in data collection at 24 months means that changes in PA levels at follow-up testing should be interpreted with caution, as the mean PA levels reflect only a sub-sample of participants. The fact that this study included only mixed gender schools means that results are delimited to this cohort, and cannot be generalised to participants attending single gender schools. Given the Y-PATH programme itself targets physical activity self-efficacy and motivation (among other factors), future research should investigate these as potential mediators through which the Y-PATH intervention exerted it’s effect on MVPA, something which was not considered in this study. Finally, although the Y-PATH intervention may be considered relatively low cost, with no personnel cost involved in rollout aside from that incurred in CPD delivery, in order to truly determine the efficacy a cost-effectiveness analysis may be warranted.

## Conclusions

Findings of this study support the efficacy of the Y-PATH intervention in helping to alleviate the age-related decline in MVPA over a two-year period in adolescent youth. Results provide further support for the efficacy of such school-based multi-component interventions. Translation of the research findings to routine practice in Irish schools is a critical next step for the intervention programme if it is to have a lasting effect on public health. The commitment of key stakeholders nationally to a sustainable national dissemination plan in this regard is a considerable step forward. Future research should investigate whether the effect of the Y-PATH intervention is specific to school day or weekend MVPA, whether the effect would remain consistent across single gender schools and with a larger sample as it is scaled up, and if the intervention would continue to have an effect beyond the two-year period.
